# P-1034. Week One Outcomes of Rezafungin vs Caspofungin Treatment for Candidemia and Invasive Candidiasis (CIC): Pooled Analysis of Two Randomized Trials Exploring Optimal Echinocandin Duration

**DOI:** 10.1093/ofid/ofae631.1224

**Published:** 2025-01-29

**Authors:** Luis Ostrosky-Zeichner, Mark A Redell, Jalal A Aram

**Affiliations:** McGovern Medical School. UTHealth, Texas, Texas; Melinta Therapeutics, Parsippany, New Jersey; Melinta Theraputics, East Lyme, Connecticut

## Abstract

**Background:**

The Phase 2 STRIVE and Phase 3 ReSTORE trials demonstrated the efficacy and safety of rezafungin for treating CIC. With data suggesting early treatment benefit for rezafungin, possibly due to its front-loaded dosing, this pooled analysis of the data assessed the outcomes of the first week of treatment in relation to later timepoints exploring the optimal duration of echinocandin therapy.

**Figure .**
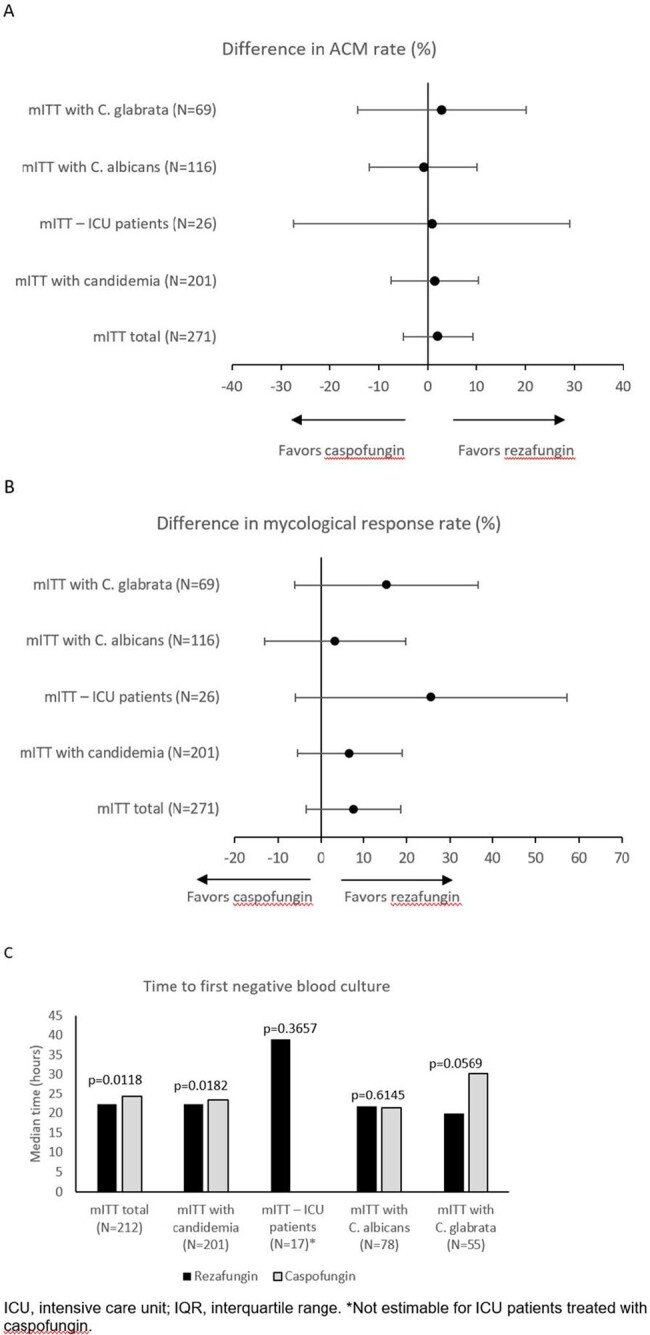
Day 7 outcomes: (A) % (95% CI) difference in ACM rate (rezafungin – caspofungin), (B) % (95% CI) difference in mycological response rate (rezafungin – caspofungin), and (C) median (IQR) TTNBC in pts with a positive blood culture before randomization (mITT population).

**Methods:**

STRIVE/ReSTORE were randomized, double-blind trials. Pts ≥18 yrs with CIC received once-a-week IV rezafungin (400 mg on Week 1, then 200 mg weekly) or once-a-day IV caspofungin (70 mg loading, then 50 mg daily) for 28 days. Efficacy was evaluated in the pooled modified intent-to-treat (mITT) population (rezafungin n=139, caspofungin n=132). Endpoints were all-cause mortality (ACM), mycological response, and time to first negative blood culture (TTNBC; in pts with a positive blood culture before randomization) at Day (D) 7, D14, and D30. Treatment-emergent adverse events (TEAEs) were assessed up to D7 in the pooled safety population (rezafungin n=151, caspofungin n=166).

**Table.**
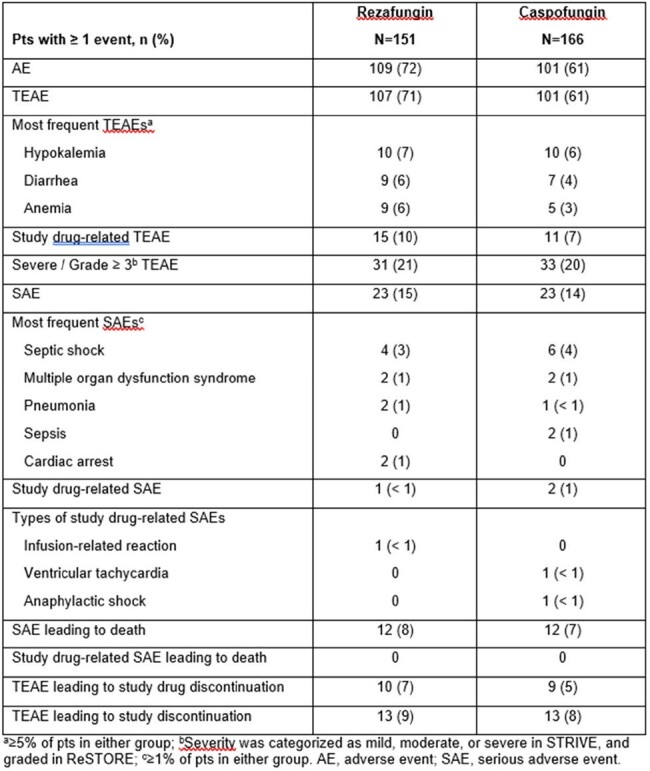
Day 7 safety (safety population)

**Results:**

Safety of rezafungin in Week 1 was comparable to its overall safety profile and the safety of caspofungin (Table). Most frequent TEAEs in Week 1 were hypokalemia, diarrhea, and anemia. ACM was similar between the two groups at D7 (% difference [95% CI] [rezafungin – caspofungin], 2.1 [-5.0, 9.2]; Figure A), D14 (1.7 [-6.4, 9.7]), and D30 (-2.3 [-12.0, 7.4]). Mycological response was also comparable between the groups at D7 (% [95% CI] difference in eradication rate, 7.6 [-3.5, 18.6]; Figure B), D14 (5.3 [-5.7, 16.2]), and D30 (7.7 [-3.6, 19.0]). TTNBC was shorter for rezafungin vs caspofungin (median, 22.3 vs 24.3 h; p< 0.05). D7 subanalyses were limited by small numbers (Figure C) but showed that TTNBC was shorter for rezafungin vs caspofungin in pts with candidemia (median, 22.3 vs 23.4 h; p=0.0182).

**Conclusion:**

Rezafungin demonstrated good safety and efficacy comparable to that for caspofungin in the first week of treatment. Rezafungin resulted in shorter TTNBC compared to caspofungin. These data provide the foundation for future investigation of the optimal duration of echinocandin therapy for invasive candidiasis.

**Disclosures:**

**Luis Ostrosky-Zeichner, MD, FACP, FIDSA, FSHEA, FECMM, CMQ**, Cidara: Advisor/Consultant|Enanta: Advisor/Consultant|F2G: Advisor/Consultant|Gilead: Advisor/Consultant|GSK: Advisor/Consultant|Melinta: Advisor/Consultant|Octapharma: Advisor/Consultant|Pfizer: Advisor/Consultant|Pfizer: Grant/Research Support|Pulmocide: Grant/Research Support|Scynexis: Grant/Research Support|Viracor: Advisor/Consultant **Mark A. Redell, PharmD**, Johnson & Johnson: Stocks/Bonds (Public Company)|Melinta Therapeutics: Full-time employee|Melinta Therapeutics: Stocks/Bonds (Private Company) **Jalal A. Aram, M.D.**, Melinta Theraputics: Employee

